# 

**Published:** 1907-02-09

**Authors:** 


### BOOKS RECEIVED.

Baillière, Tindall, and Cox.

"Surgery of the Rectum." By Fred. C. Wallis, M.D.

"Lessons on Massage." Third edition. By Margaret D. Palmer.

Longmans, Green and Co.

"The Essentials of Histology." Seventh edition. By E. A. Schäfer, LL.D.

T. Fisher Unwin.

"The Sanitary Evolution of London." By Hy. Jephson, L.C.C.

The Scientific Press, Ltd.

"A Manual for Nurses on Abdominal Surgery." By H. Burrows, M.B.

"The Local Government Journal" Office.

"The Local Government Annual and Official Directory," 1907.

Surgery Publishing Co., New York.

"Plaster of Paris, and How to Use it." By Martin W. Ware, M.D.

### Things You Must Know.

If you are a Nurse and desire to be successful and efficient you must thoroughly understand the essentials of your Profession. For instance, you must know

### HOW TO BANDAGE

**A PRACTICAL GUIDE TO SURGICAL BANDAGING AND DRESSING,** by Dr. JOHNSON SMITH, **2s.** post free, will give you all the necessary information upon this very important section of Nursing.

### NAMES OF SURGICAL INSTRUMENTS AND APPLIANCES

**SURGICAL INSTRUMENTS AND APPLIANCES USED IN OPERATIONS,** fully illustrated, with Explanatory Notes, by HAROLD BURROWS, M.B.Lond., B.S., **1s. 8d.** post free, will enable you to recognise and classify instruments required for various operations.

### THE ORGANS OF THE HUMAN BODY AND THEIR PROCESSES

**THE HUMAN BODY: ITS PERSONAL HYGIENE AND PRACTICAL PHYSIOLOGY,** by B. P. COLTON, **5s.** post free, and **ELEMENTARY PHYSIOLOGY FOR NURSES,** by C. F. MARSHALL, M.D., **2s.** post free, are useful and complete works on this subject.

### THE ANATOMY AND CONSTRUCTION OF THE BODY

**ELEMENTARY ANATOMY FOR NURSES,** by WILLIAM MCADAM ECCLES, M.S., M.B., **2s. 6d.** post free; **MUSCLES AND NERVES: AN ATLAS OF THE SUPERFICIAL MUSCLES AND PRINCIPAL MOTOR NERVES OF THE HUMAN BODY,** by LOUIS B. RAWLING, F.R.C.S., **3s. 9d.** post free. With the assistance of these works a good knowledge of the muscular elements which enter into the formation and contour of the Human Body are rapidly obtained.

### MEDICAL TERMS AND THEIR MEANINGS

**THE NURSE'S DICTIONARY OF MEDICAL TERMS AND NURSING TREATMENT,** with Pronunciation, compiled by HONNOR MORTEN, **2s.** post free, is a useful work for immediate reference, and its size will admit of it being conveniently carried in the apron pocket.

### THE WHOLE ROUTINE OF NURSING, FROM THE TAKING OF THE TEMPERATURE TO NURSING THE MOST SERIOUS DISEASES

**A HANDBOOK FOR NURSES,** by Dr. J. K. WATSON, **5s. 4d.** post free, is the most complete and up-to-date book on Nursing generally, and should occupy a prominent position on the Nurse's bookshelf. It will prove most valuable as a work of reference either for the probationer or the fully-qualified Nurse.

Many other useful books are contained in our Catalogue of Nursing Manuals, of which we shall be pleased to send a copy to any Nurse on receipt of a postcard giving name and address.

THE SCIENTIFIC PRESS, Ltd., THE NURSERY OF NURSING TEXT-BOOKS,

28 & 29 Southampton Street, Strand, London, W.C.

